# Expanding the knowledge about *Leishmania* species in wild mammals and dogs in the Brazilian savannah

**DOI:** 10.1186/s13071-015-0780-y

**Published:** 2015-03-21

**Authors:** Rebecca Martins Cardoso, Nadjar Nitz Silva Lociks de Araújo, Gustavo Adolfo Sierra Romero, Thaís Tâmara Castro Minuzzi Souza, Ana Gabriela Dietrich, Júnio Donizette Mendes, Marcelo Lima Reis, Jônatas Barbosa Cavalcante Ferreira, Mariana Machado Hecht, Rodrigo Gurgel-Gonçalves

**Affiliations:** Núcleo de Medicina Tropical, Faculdade de Medicina, Universidade de Brasília, 70904970 Distrito Federal, Brasília, Brazil; Laboratório Multidisciplinar de Pesquisa em Doença de Chagas, Faculdade de Medicina, Universidade de Brasília, 70904970 Distrito Federal, Brasília, Brazil; Laboratório de Parasitologia Médica e Biologia de Vetores, Faculdade de Medicina, Universidade de Brasília, 70904970 Distrito Federal, Brasília, Brazil; Instituto Chico Mendes de Conservação da Biodiversidade, 70670350 Distrito Federal, Brasília, Brazil

**Keywords:** *Leishmania* Eco-epidemiology, Reservoirs, Molecular diagnostics, PCR

## Abstract

**Background:**

Wild, synanthropic and domestic mammals act as hosts and/or reservoirs of several *Leishmania* spp. Studies on possible reservoirs of *Leishmania* in different areas are fundamental to understand host-parasite interactions and develop strategies for the surveillance and control of leishmaniasis. In the present study, we evaluated the *Leishmania* spp. occurrence in mammals in two conservation units and their surroundings in Brasília, Federal District (FD), Brazil.

**Methods:**

Small mammals were captured in Brasília National Park (BNP) and Contagem Biological Reserve (CBR) and dogs were sampled in residential areas in their vicinity. Skin and blood samples were evaluated by PCR using different molecular markers (D7 24Sα rRNA and rDNA ITS1). *Leishmania* species were identified by sequencing of PCR products. Dog blood samples were subjected to the rapid immunochromatographic test (DPP) for detection of anti*-Leishmania infantum* antibodies.

**Results:**

179 wild mammals were studied and 20.1% had *Leishmania* DNA successfully detected in at least one sample. Six mammal species were considered infected: *Clyomys laticeps*, *Necromys lasiurus*, *Nectomys rattus*, *Rhipidomys macrurus*, *Didelphis albiventris* and *Gracilinanus agilis.* No significant difference, comparing the proportion of individuals with *Leishmania* spp., was observed between the sampled areas and wild mammal species. Most of the positive samples were collected from the rodent *N. lasiurus*, infected by *L. amazonensis* or *L. braziliensis.* Moreover, infections by *Trypanosoma* spp. were detected in *N. lasiurus* and *G. agilis*. All 19 dog samples were positive by DPP; however, only three (15.8%) were confirmed by PCR assays. DNA sequences of ITS1 dog amplicons showed 100% identity with *L. infantum* sequence*.*

**Conclusions:**

The results suggest the participation of six species of wild mammals in the enzootic transmission of *Leishmania* spp. in FD. This is the first report of *L. amazonensis* in *N. lasiurus.*

**Electronic supplementary material:**

The online version of this article (doi:10.1186/s13071-015-0780-y) contains supplementary material, which is available to authorized users.

## Background

The leishmaniasis is caused by more than 20 different protozoan species of the genus *Leishmania* and constitutes a serious public health problem worldwide [1]. Parasites are transmitted by phlebotomine sand flies which become infected during blood meal on mammalian hosts. In Brazil, visceral leishmaniasis (VL) is present in 21 states, while the cutaneous forms of the disease (CL) were described in all states since 2003 [2]. In the Federal District of Brazil (FD), VL transmission began in 2005 in residential areas near forests. Moreover, domestic dogs infected with *Leishmania* spp. have been registered throughout the FD [3].

Several species of wild, domestic and synanthropic mammals have been recorded as hosts and/or reservoirs of *Leishmania* spp. in Brazil [4-12]. Currently, it is accepted that a reservoir may be one or a complex of species responsible for maintaining the parasite in nature in a given period and space [13,14]. The transmission of *Leishmania* species in the wild still represents a complex enzootic “puzzle”, as several links have not been identified [12].

Although dogs are considered the most important domestic reservoirs of *L. infantum* [15], the role of *Didelphis* spp. as reservoirs have been suggested and its synanthropic capability could facilitate the connection between wild and peridomestic environments [16-19]. Moreover, many rodent species have been identified as potential reservoirs of *L. amazonensis*, *L. braziliensis* and *L. infantum*, showing competence to maintain these parasites [12].

Molecular characterization of the parasites in wild reservoirs indicates the existence of different transmission cycles, illustrating the complexity of the enzootic *Leishmania* spp. transmission [12]. Then, studies of *Leishmania* spp. reservoirs in different areas are fundamental to understand host-parasite interactions and develop strategies for the surveillance and control of leishmaniasis. The identification of *Leishmania* infection in small mammals could be helpful to define the role of these animals in the transmission cycle.

The Brasília National Park (BNP) and the Contagem Biological Reserve (CBR) are federal conservation units where several species of vectors and potential reservoirs of *Leishmania* spp. could be present; however, *Leishmania* spp. enzootic transmission in the Brazilian savannah remains poorly investigated. This study aimed to detect *Leishmania* species in wild and domestic mammals, living in or near both conservation areas, and explore whether: (1) *Leishmania* species infecting wild animals at conservation units are the same species infecting domestic dogs in the vicinity; (2) there is significant difference in the frequency of *Leishmania* spp. among mammal species and between the conservation units surveyed.

## Methods

### Study area

The research was carried out in two conservation units, BNP (15°37′31”S; 47°57′36”W) and CBR (15°40′25”S; 47°51′56”W), and in houses of the surrounding areas, located in the administrative region of Sobradinho, in northern FD. CBR and BNP occupy an area of 3460 and 42389 hectares, respectively. Several phytophysiognomies are present in these conservation units such as grasslands, cerrado sensu stricto and gallery forests [20]. According to the Köppen classification the climate of the region is altitude tropical (CWa and CWb), with a cold and dry season (April-September) and a warm and rainy season (October to March). The Lago Oeste is a rural area that borders the BNP. The Grande Colorado is a region in Sobradinho formed by 10 residential areas that borders CBR, of which two were sampled, Vivendas Bela Vista (15°39′21”S; 47°51′55”W) and Mansões Colorado (15°40′18”S 47°51′21”) (Additional file 1: Figure S1).

### Animal trapping and sample collection

Mammal trapping was carried out monthly for four consecutive nights, from November 2011 to July 2012. We performed five field campaigns (Nov, Jan, Mar, May, Jul) in CBR and four (Dec, Feb, Apr, Jul) in BNP. Wild mammals were captured using Sherman and cage traps baited with a mixture of sardines, corn flour, corn, peanut butter and banana. The traps were set every 10 meters on grids in the selected areas. In CBR, we used a grid with eight parallel transects with 21 capture stations each, covering gallery forest and cerrado sensu stricto. In the BNP we used two trap grids, one in the gallery forest, formed by five parallel transects containing 11 capture stations each, and the other in the grassland type vegetation formed by 10 lines, each with 10 capture stations. In the gallery forests of CBR and BNP, the Sherman traps were placed mostly at a height of 1 to 2 m from the ground.

After capture, animals were sent to a temporary base camp, where they were contained with a combination of ketamine (Agener União, Minas Gerais) and midazolam (Eurofarma Lab. LTDA, São Paulo) at dosages of 10 to 40 mg/kg and 2 to 4 mg/kg, respectively. Whole blood samples from mammal’s retro orbital plexus were collected and transferred to filter paper. Fragments of ear skin were excised and transferred to dry Eppendorf tubes.

Animal identification was performed using taxonomic keys based on external characters [21] and species confirmation was done by a mastozoologist (MLR). Specimens were tagged in the left ear with earrings specific for small mammals (ZT 900, No. 01, ~ 7 mm) and released in their capture sites. The material was transported on the same day to the Leishmaniasis Laboratory of the Center for Tropical Medicine at University of Brasília (UnB), where they were kept at 4° C (blood) and −20° C (skin) until DNA extraction.

For convenience, dog sampling was performed in two stages. In the first stage, in June 2013, animals that presented *Leishmania-*positive results in the serological tests conducted by the environmental surveillance service in the Condominium Vivendas Bela Vista (Grande Colorado) were selected. The second sampling was performed in Condominium Mansões Colorado and in the Lago Oeste in September 2013. During the collection, an individual clinical-epidemiological form was filled with the following dog data: name, gender, age, race, color, origin, use of a repellent collar, vaccination, housing and mobility, owner’s name and address. Blood samples (3 to 5 mL) were collected by venipuncture of the cephalic or femoral vein, which were then transferred to tubes with anticoagulant (K3E Vacuette™ K3EDTA). After that, 500 μL of plasma were collected for serology and 300 μL of total blood were used for DNA extraction.

The study was approved by the Ethics Committee on Animal Use (CEUA) Institute of Biological Sciences, University of Brasília (UnBDoC No. 105819/2011) and by the Chico Mendes Institute for Biodiversity Conservation-ICMBio (authorization n ° 29486–4).

### Serology

All dog blood samples were subjected to the rapid chromatographic immunoassay dual path test using the TR DPP™ (Dual Path Platform) Canine Visceral Leishmaniasis Bio-Manguinhos Kit (Biomanguinhos, Brazil).

### PCR and sequencing

DNA from blood samples was extracted using the Wizard Genomic DNA Purification kit (Promega, USA) and DNA extraction from ear skin fragments was performed using the Illustra tissue & cells genomic Prep Mini Spin kit (GE Healthcare, USA), according to the manufacturer’s instructions. DNA samples obtained from filter papers were extracted as described by Romero et al. [22]. To evaluate endogenous quality and DNA integrity of the samples, PCR was performed for amplification of 800-bp fragment of the β-actin gene by using primers FW 5′-CGG AAC CGC TCA TTG CC-3′and 5′-ACC BW CAC ACT GTG CCC ATC TA -3′ [23].

We also amplified the polymorphic region of D7 24Sα rRNA gene (primers D75 5′-GCA GAT CTT GGT TGG CGT AG -3′ and D76 5′-GGT TCT CTG TTG CCC CTT TT -3′) that correspond to conserved sequences of trypanosome genomes, with ~ 225-bp fragments suggestive of *Leishmania* spp. [24,25]. PCR was performed on 25 μL volume containing 20 ng/μL DNA, 2.0 μM MgCl_2_, 0.2 μM dNTPs, 0.2 μM of each primer and 1.5U of Platinum Taq DNA polymerase (Invitrogen, Life Technologies, Brazil). The amplification conditions were described by Schijman et al. [26]. Positive controls used in PCR reactions were obtained from cultures of *Trypanosoma cruzi* (Berenice strain), *T. rangeli* (SC-58 strain) and *L. (L) infantum* (MCER/ BR/1979/M6445). DNA from uninfected *Mus musculus* reared in the bioterium of the Faculty of Medicine (UnB) was used as negative control. All reactions were performed in a TC-Plus thermocycler (Techne, England, UK) in triplicate. PCR products were analyzed by electrophoresis on 6% polyacrylamide gel or 2% agarose gel. Bands with molecular weight of 225-bp were considered positive.

To confirm the positive *Leishmania* spp. results we used ITS1-PCR with fragments ranging between 302 and 338-pb using primers LITS1 5′- CTG GAT TTT GCC CAT ATG - 3′ and L5.85 5′- TCG TGA TAC CAC TTA CAC TT - 3′ as described by Schönian et al*.* [27] and modified by Tojal da Silva et al. [28]. The reactions were prepared in a final volume of 25 μL containing 20 ng/μL DNA, 2.0 μM MgCl_2_, 0.2 μM dNTPs, 0.1 μM of each primer and 1.5U of Platinum Taq DNA polymerase (Invitrogen, Life Technologies, Brazil), under the following conditions: 95°C for 5 minutes, 35 cycles at 95°C for 30 seconds, 58°C for 30 seconds, 72°C for 30 seconds with a final extension step at 72°C for 5 minutes. To increase the sensitivity of the reaction, PCR products were re-amplified with the same primers and the same conditions. All reactions were performed in a TC-Plus thermocycler (Techne, England, UK) in triplicate. Positive controls used in PCR consisted of DNA from cultures of *L. (L) infantum* (MCER/BR/1979/M6445), *L. amazonensis* (IFLA/BR/1967/PH8) and *L. braziliensis* (MHOM/BR/1975/M2904). The specificity of the primers was tested with DNA extracted from uninfected *Mus musculus* reared in the bioterium of the Faculty of Medicine (UnB). Amplicons were separated on 1.3% agarose gel.

Amplified fragments were purified using Illustra GFX PCR DNA & Gel Band Purification Kit (GE Healthcare, New York, USA) and sequenced for identification of the *Leishmania* species by the companies Genomic Engenharia Molecular (São Paulo, Brazil) and CEGH-CEL (USP - São Paulo, Brazil). Sequences obtained were edited using the Geneious software (Biomatters, New Zealand) and compared with sequences deposited at GenBank using the BLASTn algorithm (Basic Local Alignment Search Tool) at the National Center for Biotechnology Information of the United States of America (http://blast.ncbi.nlm.nih.gov/Blast.cgi). The sequence data were deposited in GenBank (accession numbers KP274860 -KP274863). Fisher exact test was used to compare the proportion of *Leishmania*-positive individuals between the sampled areas and wild mammal species. Differences were considered statistically significant when p <0.05.

## Results

**Figure 1:**
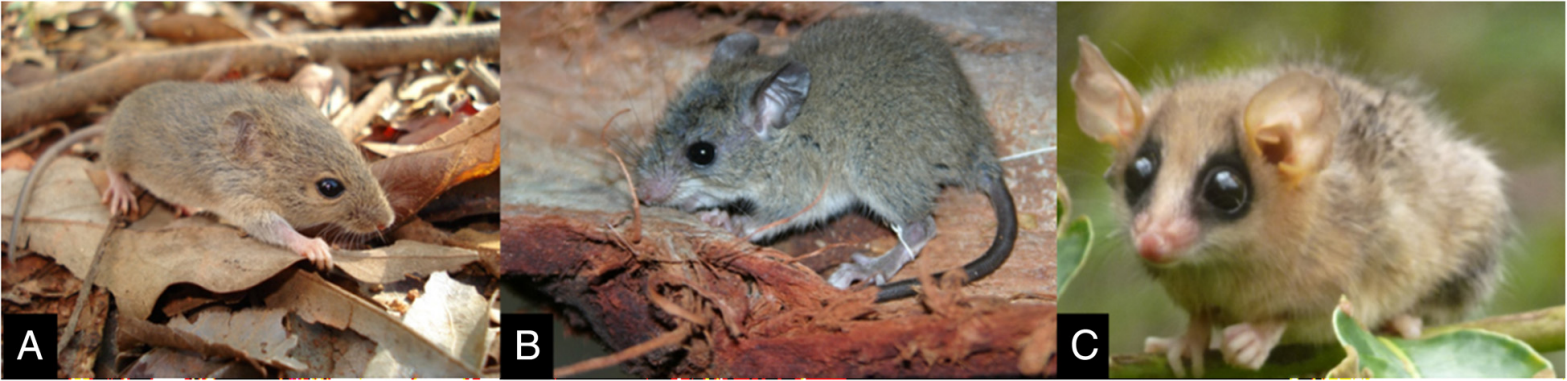
**Small mammal species with higher frequency in the study areas. A.** *Necromys lasiurus*, **B.** *Rhipidomys macrurus*; **C.** *Gracilinanus agilis.*

With a capture effort of 5,840 trap-nights, 183 individuals from 12 species of wild mammals were captured, comprising 86 individuals from 11 species captured in CBR and 97 individuals from eight species captured in the BNP. The most frequent species was *Necromys lasiurus* (Figure 1a), mainly in cerrado sensu stricto and grasslands. *Rhipidomys macrurus* (Figure 1b) and *Gracilinanus agilis* (Figure 1c) were the most frequent species in the gallery forests. The other species were captured in very low proportions (<6%) and some were found exclusively in a single area.

PCR was performed in 163 ear skin samples and 156 blood samples of 179 wild mammals, 84 captured at CBR and 95 captured at BNP (Table 1). Four samples were excluded during DNA quality control analyzes. Of the 179 samples studied, 36 (20.1%) were positive for the 24Sα rRNA gene, showing a fragment of approximately 225-bp, suggesting *Leishmania* spp., and eight (4.5%) were positive for ITS1-PCR, confirming the presence of *Leishmania* spp.

**Table 1:** **Numbers of positive blood and skin samples for the 24Sα rRNA gene of trypanosomatids and for the ITS1 of** ***Leishmania*** **spp. in individuals from conservation units of the Federal District, Brazil**

**Species**	**CBR**			**PNB**		
	**n**	**24Sα rRNA**	**ITS1**	**n**	**24Sα rRNA**	**ITS1**
**Rodentia**						
*Necromys lasiurus*	20	6	1	70	12	2
*Nectomys rattus*	8	2	1	2	0	0
*Rhipidomys macrurus*	25	7	1	1	0	0
*Oecomys bicolor*	6	0	0	0	0	0
*Cerradomys scotti*	2	0	0	0	0	0
*Thalpomys lasiotis*	2	0	0	2	0	0
*Calomys* sp*.*	1	0	0	6	0	0
*Clyomys laticeps*	0	0	0	2	1	0
**Didelphimorphia**						
*Gracilinanus agilis*	16	5	2	9	2	1
*Didelphis albiventris*	3	1	0	3	0	0
**Carnivora**						
*Nasua nasua*	1	0	0	0	0	0
Total	84	21	5	95	15	3

The species showing positive samples were *Clyomys laticeps*, *N. lasiurus*, *Nectomys rattus*, *R. macrurus*, *Didelphis albiventris* and *G. agilis.* Most of the positive samples were from the rodent *N. lasiurus* (Table 1).

No significant difference was observed when comparing the proportion of individuals with *Leishmania* spp*.* between sampled areas and among species (p > 0.05). Most of wild mammals (32/36) showed skin positive results for the 24Sα rRNA gene; three mammals (*N. lasiurus*, *G. agilis* and *R. macrurus*) were positive in skin and blood samples*.* Moreover, 7/8 ITS1 positive samples were detected only in the skin. No individual was positive for ITS1 in both samples simultaneously.

Sampled dogs, 12 males and 7 females, lived exclusively in residential areas. Eleven (58%) used insecticide impregnated collars and 13 (68%) were vaccinated against leishmaniasis. Of the 19 dogs, four (21%) showed clinical signs suggestive of leishmaniasis: weight loss, skin lesions or onychogryphosis. All samples from these dogs were positive in the DPP test. The rRNA 24Sα PCR was positive in three (15.8%) dogs, a result that was confirmed by ITS1-PCR (Additional file 2: Figure S2).

Amplicon sequencing allowed the determination of *Leishmania* species in the studied areas. ITS1-PCR identified *L. infantum* in two dogs (No. 2 and 7). *L. amazonensis* was detected in one *N. lasiurus* captured in the grassland of BNP and *L. braziliensis* in one *N. lasiurus* captured in the cerrado sensu stricto of CBR. The sequencing of the 24Sα rRNA PCR products showed sequences with 89% identity to *L. braziliensis* obtained from two *R. macrurus* captured in the gallery forest of CBR (Table 2).

**Table 2:** **Characteristics of mammals and parasites identified in two conservation units of the Federal District, Brazil**

**Individual number**	**Species**	**Location**	**Sample**	**PCR**	**Blastn** ^**1**^	**Access number**	**Identity**
2	*C. familiaris*	Bela Vista	Blood	ITS1	*L. infantum*	gb|KC347301.1	313/313(100%)
7	*C. familiaris*	Bela Vista	Blood	ITS1	*L. infantum*	gb|KC347301.1	314/314(100%)
3	*N. lasiurus*	BNP^2^	Skin	ITS1	*L. amazonensis*	gb|FJ753373.1	331/331(100%)
437	*N. lasiurus*	CBR^3^	Skin	ITS1	*L. braziliensis*	gb|FJ753379.1	300/302(99%)
479	*R. macrurus*	CBR	Skin	24Sα rRNA	*L. braziliensis*	gb|JX030149.1	153/171(89%)
481	*R. macrurus*	CBR	Skin	24Sα rRNA	*L. braziliensis*	gb|JX030097.1	153/171(89%)

1: Basic Local Alignment Search Tool. 2: Brasília National Park. 3: Contagem Biological Reserve.

Of the 179 individuals, 13 (7.3%) had samples with fragments of ~ 240-pb to 290-bp of the 24Sα rRNA gene, suggesting positive infection by *Trypanosoma* spp. However, no blood sample was positive to *T. cruzi* using specific primers TCZ1/2 (data not shown). Sequences analyses of the 24Sα-rRNA PCR products of three individuals of *N. lasiurus* showed between 98% and 99% identity with *T. otospermophili* and 98% identity with *T. kuseli* and *T.* g*rosi.* Moreover, DNA sequence of a *G. agilis* had 91% identity with *T. grosi.* The sequencing of the ITS1-PCR products of three *N. lasiurus* showed an identity of 79% and 82% with *T. otospermophili.* Other specimen of *N. lasiurus* showed 79% identity with *T. lewisi*.

## Discussion

DNA of three *Leishmania* species was detected in wild mammals of the conservation units and domestic dogs from the surrounding areas*.* Of interest, *Leishmania* species in wild animals within the conservation units (*L. amazonensis* and *L. braziliensis*) were not the same from the species detected in domestic dogs (*L. infantum*)*.* This fact could indicate that i) there are different and independent cycles of *Leishmania* species in the studied areas; ii) *L. infantum* has not established yet a sylvatic transmission cycle in the area being maintained by dogs and maybe other synanthropic mammals (e.g. *Didelphis albiventris*); iii) although the results do not define the reservoir of *L. braziliensis* in the area, they are sufficient to point out that *L. braziliensis* includes in its transmission cycle the rodent species *N. lasiurus*. However, sampling effort for dogs was small and we cannot exclude the possibility of *L. braziliensis* or *L. amazonensis* circulating within canine population as reported in other endemic areas for these species [29,30].

Dogs have a fundamental role in the domestic cycle of *L. infantum* because they are highly susceptible to infection, carry an intense, long lasting parasite infection and live in or near human habitations [9,31,32]. Moreover, dogs are proven sources of infection to sand flies due to the abundant amastigotes in their skin which facilitates *L. infantum* transmission [33]. Wild mammals, however, favor the maintenance of *L. amazonensis* and *L. braziliensis* in the conservation units. *Leishmania amazonensis* was identified with 100% identity in the DNA extracted from the skin sample of one *N. lasiurus* captured in grasslands at the BNP. This is the first record of *L. amazonensis* in this rodent species which shows a wide potential distribution in Brazil [34] and is abundant in open and dry areas of Brazilian savannah [35,36]. Moreover, our data suggest that *R. macrurus* is a possible host of *L. braziliensis.* Until now, there was only the record of *R. mastacalis* infected with *L. infantum* in Minas Gerais [10]. The results suggest potential participation of at least six species of different genera of wild mammals in the biological cycle of *Leishmania* spp. in FD. All these six genera were already associated with *Leishmania* spp. [7-10,12,37].

The present study focused on wild mammals in conservation units, but there is a need for further studies with synanthropic mammals (e.g. *D. albiventris*)*.* The role of wild mammals as a source of infection for peridomiciliary vectors should not be ignored [12], since the participation of these mammals in the transmission cycle of *L. infantum* in urban areas has been proposed [17-19].

Although no statistical difference was detected in the proportion of *Leishmania*-positive mammals in the different areas, the CBR showed a higher rate of positive samples. CBR has increased anthropogenic influence with the establishment of residential areas around it and with a heavy flow of pets and people within it. Future longitudinal studies analyzing a larger number of mammals and including data of the sand fly infection in these areas may explain the effect of environmental changes in the enzootic transmission of *Leishmania.*

*Necromys lasiurus* was the most prevalent mammal species in this study (50.28%) with 20% of the samples positive for the 24Sα rRNA gene. This rodent species is considered a potential reservoir because of its role in the maintenance of infection and the potential to transmit the parasite to the vectors [38,39]. The infection rate of species ranged from 16.7 to 28%, considering the species with higher number of analyzed samples. However, no significant differences were observed when comparing the proportion of individuals with *Leishmania* spp*.* between species. The possibility of at least six species of different genera of wild mammals participating in the biological cycle of *Leishmania* spp. in the studied areas illustrates the complexity of the enzootic cycles of these parasites. Each host species should have a distinct role in the transmission of *Leishmania* according to different spatial and temporal scenarios [12]. In this study we present evidence for the association of *L. amazonensis* with cursorial rodents (*N. lasiurus*) in grasslands in the BNP and of *L. braziliensis* with *N. lasiurus* in cerrado sensu stricto in CBR. Future studies including methods that evaluate the parasitaemia and intensity of skin parasitism in these animals using quantitative approaches (qPCR) may reveal whether they are hosts of maintenance or amplification [12,39]. Experimental studies may evaluate the putative role of *N. lasiurus* and *R. macrurus* in the retention of infection and amplification of *Leishmania* spp., as already observed for other rodent species such as *Thrichomys laurentius* [40]. In addition, researches on natural infection of sand flies as well as molecular characterization of *Leishmania* species may provide more data to understand the transmission dynamics in these areas.

Positive amplification of 24Sα rRNA suggestive for *Leishmania* spp. (20.1%) was similar to the positivity of *Leishmania* infection in other studies with small mammals [10,11,37,41]. However, with more specific primers (ITS1), positivity decreased to 4.5%. Differences in rates of infection with *Leishmania* spp. among studies may be influenced by methodological differences, such as number and species of mammals sampled, protocols and PCR target used. Amplification of the polymorphic region of the D7 24Sα rRNA gene corresponding to conserved sequences of trypanosomatid genomes have different molecular weights to *Leishmania* spp. and *Trypanosoma* spp. [24-26] and it is more sensitive when compared to other targets*.* It is also used for the differential diagnosis of infection by diverse trypanosomes in reservoirs in endemic areas. ITS1-PCR has lower sensitivity compared with the highly abundant target at the kDNA conserved region, as shown in a study by Tojal da Silva et al. [28] with DNA extracted from skin biopsies from humans infected by *Leishmania* spp. In another study using human and dog blood, the SSU rDNA-PCR was more sensitive than ITS1-PCR [28]. However, when *nested* ITS1-PCR is performed, the sensitivity increases considerably and can match the other targets [27,42].

Our results also demonstrate the presence of *Trypanosoma* spp. in wild rodents and marsupials in the FD. DNA sequences extracted from samples of four *N. lasiurus* and one *G. agilis* showed identity ranging from 79-99% with *T. lewisi*, *T. grosi*, *T. otospermophili* and *T. kuseli*. Of interest, all of them are rodent trypanosomes [43-46]. *Trypanosoma lewisi* is a common blood parasite of *Rattus* spp. in all parts of the world, transmitted by fleas and *T. grosi* shows a host-restriction to mice of the genus *Apodemus*, distributed in Europe and Asia [43,44]. *Trypanosoma otospermophili* and *T. kuseli* are trypanosomes of squirrels also transmitted by fleas occurring both in old and new worlds [43,45]. The identification of these trypanosomes needs confirmation because most samples showed identity values <90% and we have no morphological evidence to support these results. It is possible that these trypanosomes belong to other species such as *T. forattinii* and *T. akodoni* detected in rodents of the genera *Oryzomys* and *Akodon* in Brazil, respectively [43], which are not deposited in GenBank. Although these protozoa are host-specific and not pathogenic to humans, studies show that at least *T. lewisi* can play a role as an opportunistic parasite in humans and captive monkeys [47].

## Conclusions

These results suggest the participation of wild mammals in the cycle of *Leishmania* spp. in conservation units of the Federal District of Brazil, expanding knowledge on the enzootic cycles of trypanosomatids in the Brazilian savannah. *Leishmania* species infecting wild animals at conservation units were not the same species infecting domestic dogs in the vicinity. Although no significant differences were observed in the infection rates among mammal species surveyed, the study highlights the role of *N. lasiurus*, *R. macrurus* and *G. agilis* as hosts of *Leishmania* spp. However, studies monitoring infection with other parasitological methods (e.g. skin and blood cultures) and sensitive molecular techniques (e.g. qPCR or nested PCR) [42], over a longer period of time including an increased sample size is required to determine whether these mammals meet the established criteria [48,49] to be considered reservoirs of these parasites and evaluate the complexity of host-parasite interactions.

## Additional files

Additional file 1: Figure S1. Study areas in Contagem biological reserve (CBR, cerrado and gallery forests) and Brasilia National Park (BNP, grassland and gallery forests), where small mammals were sampled. Stars show residential areas where dogs were sampled: Lago Oeste (red), Vivendas Bela Vista (green) and Mansões Colorado (yellow). Modified after Unesco [50] and Google earth. Additional file 2: Figure S2. Identification of *Leishmania* spp. DNA in dog samples using different molecular markers. Polyacrylamide gel (6%) showing PCR results. (A) directed to the 24Sα rRNA gene, with a fragment size of approximately 225-bp, suggestive of *Leishmania* spp., on canine samples 1 to 19, and (B) ITS1 with a fragment of approximately 310-bp of the canine samples that showed bands with the D75 and D76 primers. MW: molecular weight marker; Positive controls: *T. cruzi-* Tc, *T. rangeli-* Tr, *L. infantum-* Li, *L. braziliensis-* Lb and *L. amazonensis- La*; negative control: NC: negative control; WC: White Controls. Red arrows indicate positive samples.

## Abbreviations

CRB: Contagem Biological Reserve
PNB: Brasília National Park
PCR: Polymerase Chain Reaction
qPCR: Quantitative real-time PCR
VL: Visceral leishmaniasis
CL: Cutaneous leishmaniasis
FD: Federal District
kDNA: Kinetoplast DNA
DPP: Dual Path Platform.
