# Supplementary figures and images for: Expanding the knowledge about Leishmania species in wild mammals and dogs in the Brazilian savannah
Source: Parasit Vectors. 2015 Mar 21;8:171. doi: 10.1186/s13071-015-0780-y (PMC4386096; doi:10.1186/s13071-015-0780-y)

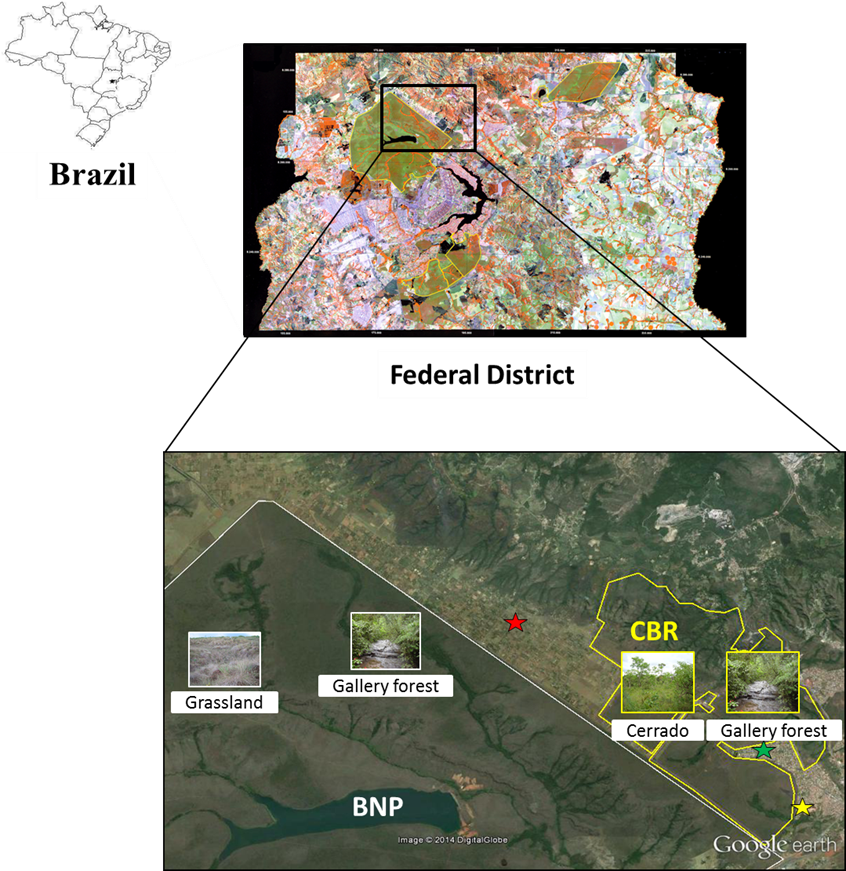

Supplement: Additional file 1: Figure S1. — Study areas in Contagem biological reserve (CBR, cerrado and gallery forests) and Brasilia National Park (BNP, grassland and gallery forests), where small mammals were sampled. Stars show residential areas where dogs were sampled: Lago Oeste (red), Vivendas Bela Vista (green) and Mansões Colorado (yellow). Modified after Unesco [50] and Google earth. [file 13071_2015_780_MOESM1_ESM.tif]

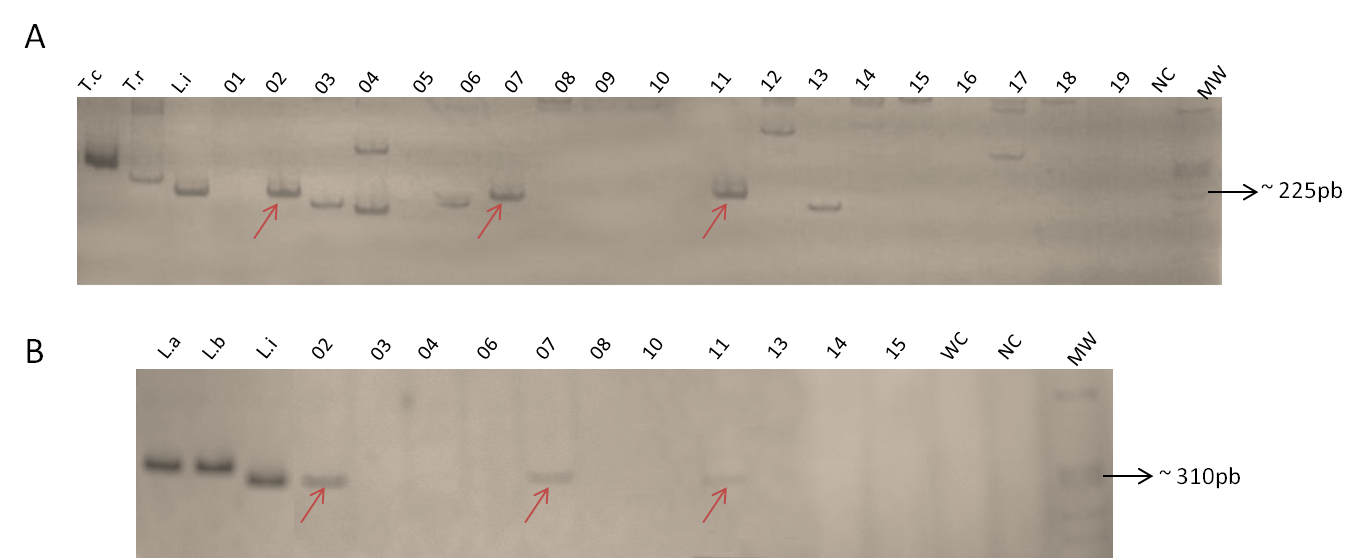

Supplement: Additional file 2: Figure S2. — Identification of Leishmania spp. DNA in dog samples using different molecular markers. Polyacrylamide gel (6%) showing PCR results. (A) directed to the 24Sα rRNA gene, with a fragment size of approximately 225-bp, suggestive of Leishmania spp., on canine samples 1 to 19, and (B) ITS1 with a fragment of approximately 310-bp of the canine samples that showed bands with the D75 and D76 primers. MW: molecular weight marker; Positive controls: T. cruzi- Tc, T. rangeli- Tr, L. infantum- Li, L. braziliensis- Lb and L. amazonensis- La; negative control: NC: negative control; WC: White Controls. Red arrows indicate positive samples. [file 13071_2015_780_MOESM2_ESM.tif]
